# α2-COP is involved in early secretory traffic in Arabidopsis and is required for plant growth

**DOI:** 10.1093/jxb/erw446

**Published:** 2016-12-26

**Authors:** Fátima Gimeno-Ferrer, Noelia Pastor-Cantizano, César Bernat-Silvestre, Pilar Selvi-Martínez, Francisco Vera-Sirera, Caiji Gao, Miguel Angel Perez-Amador, Liwen Jiang, Fernando Aniento, María Jesús Marcote

**Affiliations:** 1 Departamento de Bioquímica y Biología Molecular, Facultad de Farmacia, Universitat de València, E-46100 Burjassot, Spain; 2 Estructura de Recerca Interdisciplinar en Biotecnología i Biomedicina (ERI BIOTECMED), Universitat de València, E-46100 Burjassot, Spain; 3 Instituto de Biología Molecular y Celular de Plantas (IBMCP), Universidad Politécnica de Valencia–Consejo Superior de Investigaciones Científicas (CSIC), E-46022 Valencia, Spain; 4 School of Life Sciences, Centre for Cell and Developmental Biology and State Key Laboratory of Agrobiotechnology, The Chinese University of Hong Kong, Hong Kong, China

**Keywords:** α1-COP, α2-COP, Arabidopsis, coat protein, COPI, COPII, Golgi apparatus, p24 family protein, SEC31.

## Abstract

COP (coat protein) I-coated vesicles mediate intra-Golgi transport and retrograde transport from the Golgi to the endoplasmic reticulum. These vesicles form through the action of the small GTPase ADP-ribosylation factor 1 (ARF1) and the COPI heptameric protein complex (coatomer), which consists of seven subunits (α-, β-, β′-, γ-, δ-, ε- and ζ-COP). In contrast to mammals and yeast, several isoforms for coatomer subunits, with the exception of γ and δ, have been identified in Arabidopsis. To understand the role of COPI proteins in plant biology, we have identified and characterized a loss-of-function mutant of α2-COP, an Arabidopsis α-COP isoform. The *α2-cop* mutant displayed defects in plant growth, including small rosettes, stems and roots and mislocalization of p24δ5, a protein of the p24 family containing a C-terminal dilysine motif involved in COPI binding. The *α2-cop* mutant also exhibited abnormal morphology of the Golgi apparatus. Global expression analysis of the *α2-cop* mutant revealed altered expression of plant cell wall-associated genes. In addition, a strong upregulation of *SEC31A*, which encodes a subunit of the COPII coat, was observed in the *α2-cop* mutant; this also occurs in a mutant of a gene upstream of COPI assembly, *GNL1*, which encodes an ARF-guanine nucleotide exchange factor (GEF). These findings suggest that loss of *α2-COP* affects the expression of secretory pathway genes.

## Highlight

In Arabidopsis α2-COP is required for plant growth, Golgi structure and subcellular localization of p24δ5, with its loss of function resulting in upregulation of the COPII subunit *SEC31A*.

## Introduction

The conventional secretory pathway in plants involves the transport of newly synthesized proteins from the endoplasmic reticulum (ER) to the Golgi apparatus and then to the cell surface or vacuole. The so-called ‘early secretory pathway’ involves bidirectional transport between the ER and the Golgi apparatus, which is mediated by COP (coat protein) I and COPII vesicles (Brandizzi and Barlowe, 2013). COPII vesicles are involved in protein export from the ER, whereas COPI vesicles are involved in intra-Golgi transport, where their directionality is still a matter of debate, and in retrograde transport from the Golgi to the ER. Coat proteins are involved in the selective capture of cargo proteins within the donor compartment, including the fusion machinery needed to ensure vesicle delivery, and the generation of membrane curvature to drive vesicle formation. COPI vesicles are formed at the Golgi apparatus and facilitate the retrieval of ER resident proteins from the Golgi to the ER as well as cycling of proteins between ER and the Golgi apparatus. Many type I transmembrane proteins transported by COPI vesicles bear a C-terminal dilysine-based motif, which has been proven to be recognized by the COPI coat (Jackson *et al.* 2012).

The key component of the COPI coat is the coatomer complex, which is essential in eukaryotes and is recruited *en bloc* onto Golgi membranes (Hara-Kuge *et al.*, 1994). It is composed of seven subunits (α/β/β’/γ/δ/ε/ζ) that have been conceptually grouped into two subcomplexes, the B- (α/β ′/ε) and F-subcomplex (β/δ/γ/ζ). The B-subcomplex has been proposed to function as the outer layer and the F-subcomplex as the inner layer of the vesicle coat (Jackson, 2014). However, recent structural studies revealed that the subunits are highly connected to each other, suggesting that the COPI structure does not fit with the adaptor F-subcomplex and cage B-subcomplex structure described for other coats (Dodonova *et al.*, 2015). Following recruitment by the small GTPase ADP-ribosylation factor 1 (ARF1), in its GTP-bound conformation, and uptake of cargo, COPI polymerizes on the membrane surface in such a way that COPI coat assembly depends on both membrane and cargo binding. Several studies indicate that the β′-COP and α-COP subunits are involved in binding of cargo (i.e. proteins with a dilysine motif) through their N-terminal WD repeat domains. It has also been reported that the γ subunit interacts with ARF1 and that ζ-COP is required for the stability of γ-COP (Jackson, 2014).

Genes encoding the components of the COPI machinery have been identified in plants (Robinson *et al.*, 2007; Gao *et al.*, 2014; Ahn *et al.*, 2015; Woo *et al.*, 2015). In Arabidopsis, several isoforms of all the coatomer subunits, except for γ-COP and δ-COP subunits, have been identified. This is in contrast to mammals, where only γ-COP and ζ-COP subunits have more than one isoform, and to yeast that contains only one isoform for every subunit. Interestingly, electron tomography studies in Arabidopsis have identified two structurally distinct types of COPI vesicles (Donohoe *et al.*, 2007; Gao *et al.*, 2014). These different subpopulations of COPI vesicles might be formed by different coatomer isoforms. It is therefore of great interest to know whether these different COPI subunit isoforms have specific biological functions in plants by means of their functional characterization. Recently, the subcellular localization, protein interactions and physiological functions of β ′-, γ-, and δ-COP subunits were investigated in *Nicotiana benthamiana* and tobacco BY-2 cells. It was shown that the COPI complex is involved in Golgi maintenance and cell plate formation, and that programmed cell death is induced after prolonged COPI depletion (Ahn *et al.*, 2015). In Arabidopsis, knockdown of ε-COP subunit isoforms has been reported to cause severe morphological changes in the Golgi apparatus and mislocalization of endomembrane proteins (EMPs) containing the KXD/E COPI interaction motif (Woo *et al.*, 2015). Here, we have used a loss-of-function approach to characterize the Arabidopsis α2-COP isoform. Two α-COP isoforms, α1-COP (At1g62020) and α2-COP (At2g21390), have been identified in Arabidopsis and both isoforms contain an N-terminal WD40 domain that may allow them to recognize C-terminal dilysine-based motifs of COPI cargo proteins (Eugster *et al.*, 2004; Jackson *et al.*, 2012). We found that a loss-of-function mutant of *α2-COP* showed defects in growth. In addition, in the *α2-cop* mutant, the morphology of the Golgi apparatus was altered as was the subcellular localization of p24δ5, a protein with a dilysine motif that has been shown to cycle between the ER and the Golgi. A transcriptomic analysis of the *α2-cop* mutant showed upregulation of plant cell wall and endomembrane system genes, such as the COPII component *SEC31A*, indicating that *α2-COP* loss of function affects the expression of secretory pathway genes.

## Materials and methods

### Plant material

*Arabidopsis thaliana* ecotype Col-0 was used as wild type. The loss-of-function mutants *α1-cop-1* (SALK_078465), *α2-cop-1* (SALK_103968), *α2-cop-2* (SALK_ 1229034), and *gnl1* (SALK_091078C) were from the Salk Institute Genomic Analysis Laboratory (http://signal.salk.edu/cgi-bin/tdnaexpress). *α2-cop-3* (GABI_894A06) was from GABI-Kat (Kleinboelting *et al.*, 2012). All the mutants were obtained from the Nottingham Arabidopsis Stock Centre. *A. thaliana* plants were grown in growth chambers as previously described (Ortiz-Masia *et al.*, 2007). Lines in a Col-0 background containing a T-DNA insertion were characterized by PCR. The primers used are included in Supplementary Table S1 at *JXB* online.

### Recombinant plasmid production, plant transformation and transformant selection

The coding sequence of *α2-COP* with one human influenza hemagglutinin (HA) tag before the stop codon was synthesized commercially *de novo* (Geneart AG) based on the sequence of *α2-COP* (At2g21390). The coding sequence of *α2-COP-HA* was cloned into the pCHF3 vector carrying the CaMV 35S promoter (Ortiz-Masia *et al.*, 2007) through SmaI/SalI. To complement growth defects, *α2-cop-3* plants were transformed with the *α2-COP-HA* construct via Agrobacterium using the floral deep method according to standard procedures (Clough and Bent, 1998). To estimate the number of T-DNA insertions in the transgenic plants, 40 seeds of each T1 line were plated on 1/2 x Murashige Skoog (MS) basal salts, 1% sucrose, 0.6% agar with 50 mg/l kanamycin. Only lines where the proportion of kanamycin resistant to sensitive plants in their progeny fitted a 3:1 ratio were considered. Homozygous *α2-COP-HA* T2 lines were identified by the same method.

For confocal studies, *α2-cop-3* and wild type plants were transformed with a sialyl transferase (ST)-YFP construct (kindly provided by Dr DG Robinson, Heidelberg, Germany) and a RFP-p24δ5 construct, as described above. The RFP-p24δ5 construct was obtained by subcloning the RFP-p24δ5 coding sequence (Langhans *et al.*, 2008) in the pCHF3 vector through KpnI/BamHI. The transformants were selected with antibiotic as above. We obtained three independent ST-YFP- and RFP-p24δ5-*α2-cop-3* lines that showed the same confocal phenotypes over subsequent generations.

### Reverse Transcription PCR (RT-PCR)

Total RNA was extracted from seedlings using a Qiagen RNeasy plant mini kit and 3 μg of the RNA solution were reverse transcribed using the maxima first strand cDNA synthesis kit for quantitative RT-PCR (Fermentas) according to the manufacturer’s instructions. Semi-quantitative PCRs (sqPCRs) were performed on 3 μL cDNA template using the PCR Master kit (Roche). The sequences of the primers used for PCR amplifications are included in Supplementary Table S1.

Quantitative PCR (qPCR) was performed using StepOne Plus from Applied Biosystems with SYBR Premix Ex Taq TM (Tli RNaseH Plus) from Takara Bio according to the manufacturer’s protocol. Each reaction was performed in triplicate with 100ng of the first strand cDNA and 0.9 μM primers for *SEC31A* or 0.3 µM of primers for all other genes in a total volume of 20μl. Data are the mean of two biological samples. The specificity of the PCR amplification was confirmed with a heat dissociation curve from 60°C to 95°C. Relative mRNA abundance was calculated using the comparative Ct method according to Pfaffl (2004). Primers used for qPCR are listed in Supplementary Table S1.

### Preparation of protein extracts and western blotting

Seven-day-old seedlings were ground in liquid nitrogen and homogenized in lysis buffer, 50 mM TRIS-HCl pH 7.5, 150 mM NaCl, 0.5 mM DTT, 0.5% Triton X-100, and a cocktail of protease inhibitors (1:250 dilution, Sigma #P9599) for 30 min at 4°C. Samples were centrifuged twice at 12,000g for 20 min at 4°C and supernatants were considered as protein extracts. Protein quantitation was performed with the Bradford assay (Bio-Rad Laboratories GmbH, Munich, Germany). Protein samples were separated by electrophoresis on an 8% SDS-polyacrylamide gel and transferred to nitrocellulose membranes (Schleicher & Schuell). Before blotting, membranes were stained with Ponceau S solution (Sigma) to show loading of the protein samples. Membranes were probed with the primary antibody anti-HA High Affinity (1:500 dilution, Roche), Anti-human GAPC (1:1000 dilution, Santa Cruz) or anti-α-COP [1:2000 dilution) (Gerich *et al.*, 1995)]; and developed by enhanced chemiluminescence (ECL) (GE Healthcare) as previously described (Montesinos *et al.*, 2013). Western blots were analyzed using the ChemiDoc XRS+ imaging system (Bio-Rad, http://www.bio-rad.com/).

### Microarrays

Four-day-old seedlings of *α2-cop-3* and wild type grown in 1/2 MS plates were used. Total RNA from four pools of seedlings were extracted using the RNeasy plant mini kit (Qiagen), and RNA integrity was tested by 2100 Bioanalyzer (Agilent). The four replicas of wild type were pooled together to generate a unique reference sample that was tested against each of the individual *α2-cop-3* samples to generate four biologically independent assays, using the two-color dye-swap microarray. RNA labeling and microarray details were as described in Vera-Sirera *et al.* (2015). 0.5μg of RNA per sample was amplified and labelled with the Agilent Low Input Quick Amp Labelling Kit. Agilent Arabidopsis (V4) Gene Expression 4344K Microarrays were used. Hybridization and slide washing were carried out with the Gene Expression Hybridization Kit and Gene Expression Wash Buffers. Slides were scanned in an Agilent G2565AA microarray scanner at 5 µm resolution in a dual scan for high dynamic range. Image files were analyzed with the Feature Extraction software 9.5.1. Raw microarray data (accession number GSE81049) were deposited in the Gene Expression Omnibus (GEO). Inter-array analysis were performed with the GeneSpring 11.5 software. Only features for which the ‘r or/and gIsWellAboveBG’ parameter was 1 in at least three out of four replicas were selected. T-test analysis was carried out with Benjamin-Hochberg metrics to identify significantly expressed genes with a p-value below 0.05 after correction for multiple testing and where the expression ratio was above or below a two-fold difference (Log_2_±1). Features were converted into genes based on a BLAST analysis extracted from ftp://ftp.arabidopsis.org/Microarrays/Agilent/; oligo probes that did not correspond to any gene or to more than one gene were removed for further analysis. Gene ontology (GO) analysis at the biological process level was carried out with agriGO (http://bioinfo.cau.edu.cn/agriGO/;Du *et al.*, 2010). Only GO terms with corrected a p-value less than or equal to 0.05 were selected.

### Transmission electron microscopy

For electron microscopy, seedlings were grown on MS medium containing 1% agar and harvested after 4 days. Chemical fixation of cotyledons was performed according to Osterrieder *et al.* (2010). Ultrathin 70 nm sections were cut on a Microtome Leica UC6, stained with uranyl acetate and lead citrate and observed with a JEM-1010 (JEOL) transmission electron microscope. High pressure freezing was performed as described previously (Tse *et al.*, 2004; Gao *et al.*, 2012) and samples were analyzed in a Hitachi H-7650 transmission electron microscope.

### Confocal microscopy

Imaging was performed using an Olympus FV1000 confocal microscope (http://www.olympus.com/) with a 60x water lens. Fluorescence signals for YFP (514 nm/529–550 nm) and RFP (543 nm/593–636 nm) were detected. Sequential scanning was used to avoid any interference between fluorescence channels. Post-acquisition image processing was performed using the fv10-asw 3.1 Viewer and coreldrawx4 (14.0.0.567) or ImageJ (version 1.45 m) (Abramoff *et al.*, 2004).

## Results

### *α2-cop* mutants display a dwarf phenotype

**Fig. 1.**
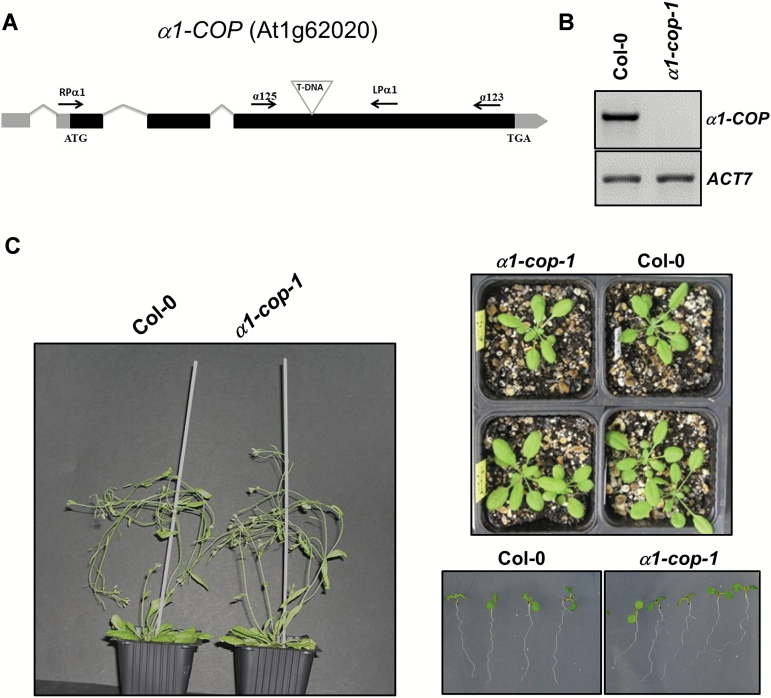
Characterization of the *α1-cop-1* mutant. A. Diagram of the *α1-COP* gene and localization of the T-DNA insertion (triangle) in the *α1-cop-1* mutant. Black boxes represent coding regions and grey boxes represent 5’ UTR and 3’ UTR regions. The positions of RPα1, LPα1, α125 and α123 primers are shown. B. sqRT-PCR analysis to show the absence of *α1-COP* mRNA in the *α1-cop-1* mutant. Total RNA from 7-day-old seedlings of the mutant and wild type (Col-0) were used for the RT-PCR. For PCR, *α1-COP* gene specific primers, RPα1/LPα1, were used with 36 PCR cycles. *ACT7* was used as a control with 22 cycles. C. *α1-cop-1* mutant did not show a phenotype different from wild type.

To investigate the function of the two isoforms of the α-COP subunit in Arabidopsis, T-DNA insertion mutants were identified and analyzed. A mutant of α1-COP, *α1-cop-1,* which had the insertion in the third coding exon, was identified in the Salk collection, corresponding to stock number SALK_078465 (Fig. 1A). Although truncated transcripts were detected (see Supplementary Fig. S1), RT-PCR analysis confirmed that this mutant lacked the full length α1-COP transcript (Fig. 1B). As shown in Fig. 1C, the *α1-cop-1* mutation did not compromise plant growth under standard growth conditions.

**Fig. 2.**
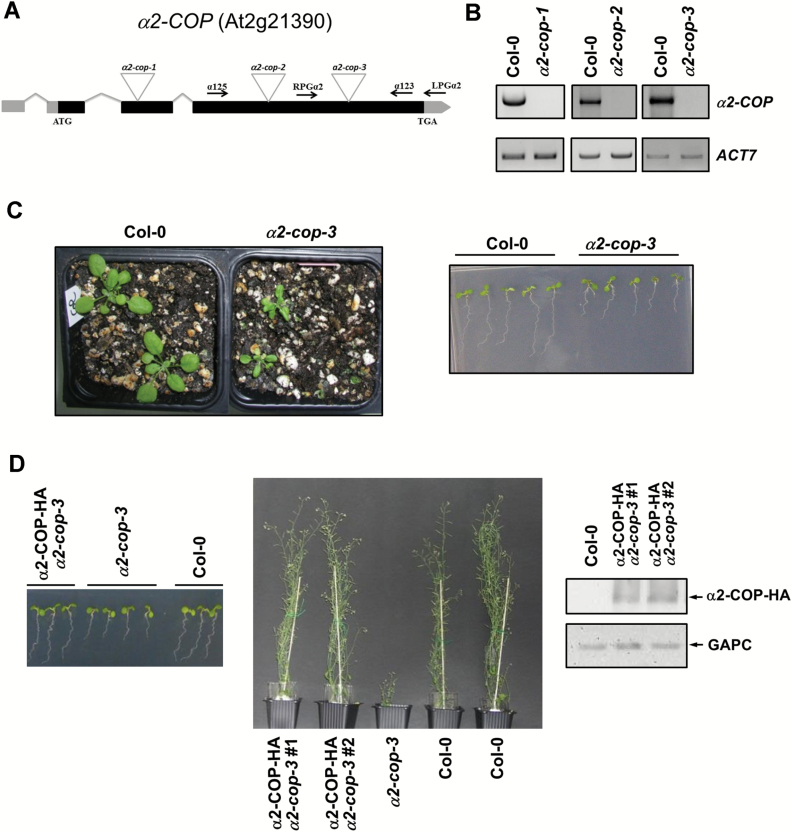
Characterization of *α2-cop* mutants. A. Diagram of the *α2-COP* gene and localization of the T-DNA insertion (triangles) in the *α2-cop* mutants. Black boxes represent coding regions and grey boxes represent 5’ UTR and 3’ UTR regions.The positions of RPGα2, LPGα2, α125 and α123 primers are shown. B. sqRT-PCR analysis to show the absence of *α2-COP* mRNA in the *α2-cop* mutants. Total RNA from 7-day-old seedlings of the mutants and wild type (Col-0) were used for the RT-PCR. For PCRs, gene specific primers and 36 PCR cycles were used (Supplementary Table S1). *ACT7* was used as a control with 22 PCR cycles. C. Phenotype of 4-week-old (left) and 7-day-old (right) seedlings of wild type and *α2-cop-3* mutant. D. Rescue of the growth phenotype of *α2-cop-3* by transformation with a HA tagged *α2-COP* cDNA construct. Phenotypes of 7-day-old seedlings (left) and 50-day-old plants (middle) of wild-type (Col-0), *α2-cop-3* and *α2-cop-3* complemented with α2-COP-HA. Western blot analysis, using a HA antibody, of the two independent lines of *α2-cop-3* transformed with α2-COP-HA.

Three mutants of α2-COP, *α2-cop-1* (SALK_103968), *α2-cop-2* (SALK_ 1229034) and *α2-cop-3* (GABI_894A06), which had the insertion in different gene positions (Fig. 2A) were characterized. Homozygous plants were selected and RT-PCR analysis confirmed that *α2-cop-1*, *α2-cop-2* and *α2-cop-3* mutants lacked the full length α2-COP transcript (Fig. 2B). As occurred in the *α1-cop-1* mutant, truncated transcripts were also detected in the *α2-cop* mutants (see Supplementary Fig. S1). In contrast to the normal growth of *α1-cop-1,* all *α2-cop* mutants exhibited dwarf phenotypes with reduced rosette and leaf size and shorter stems and roots, although they were all fertile (Fig. 2C, Supplementary Fig. S2).

Here we focused on the characterization of the *α2-cop-3* mutant for further analysis of *α2-COP* loss of function (Fig. 2). To confirm that the developmental defects in *α2-cop-3* were indeed caused by the loss of α2-COP function, we transformed these mutants with α2-COP cDNA containing a HA tag. As shown in Fig. 2D, the expression of α2-COP-HA in the *α2-cop-3* mutant fully rescued its developmental defects. These results indicate that α2-COP may be required for normal plant growth and development.

**Fig. 3.**
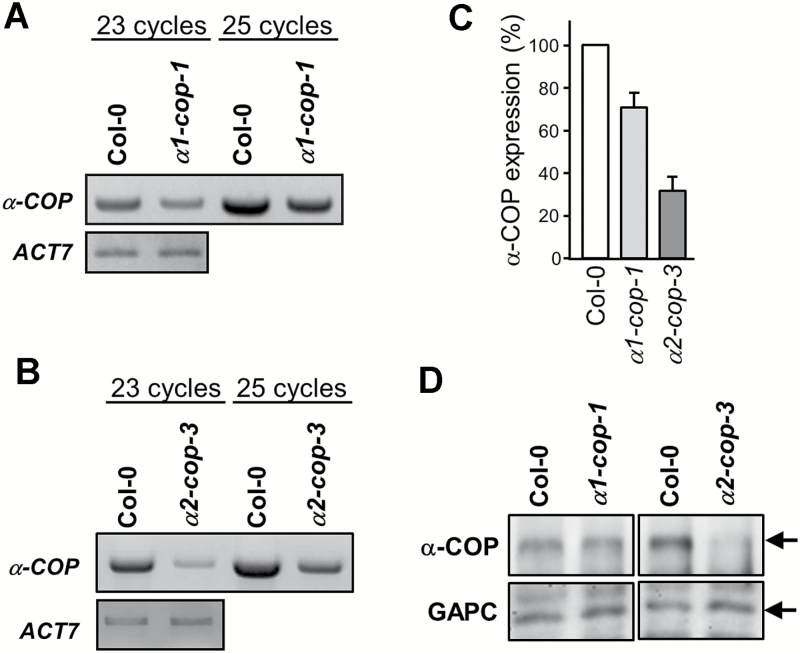
Expression levels of *α-COP* in *α1-cop-1* and *α2-cop-3* mutants. sqRT-PCR analysis of *α-COP* with α125 and α123 primers. Total RNA was isolated from 7-day-old seedlings of wild type (Col-0), *α1-cop-1* and *α2-cop-3* mutants. *ACT7* was used as a control with 22 PCR cycles. A. Total *α-COP* expression in wild type and *α1-cop-1* mutant. B. Total *α-COP* expression in wild type and *α2-cop-3* mutant. C. Quantification of the experiments shown in A and B from three biological samples. Values were normalized against the *α-COP* fragment band intensity in wild type that was considered to be 100%. Error bars represent SEM. D. Western blot analysis of total protein extracts from 7-day-old seedlings of wild type, *α1-cop-1* and *α2-cop-3* mutants using an N-terminal α-COP peptide antibody to detect both isoforms. 10 μg of total protein was loaded in each lane. GAPC was used as a loading control.

Next, the expression levels of total *α-COP* in *α1-cop* and *α2-cop* mutants were analyzed compared to wild type, in order to investigate whether the dwarf phenotype of *α2-cop* was due to lower expression levels of *α-COP*. To this end, the expression levels of total *α-COP*, including isoforms *α1* and *α2*, were analyzed by RT-PCR using a pair of primers (α125 and α123) common to *α1-COP* and *α2-COP* genes (Fig. 1, 2) that can therefore amplify the cDNA of both genes in wild type plants. However, these primers can only amplify the *α2-COP* cDNA fragment and not the *α1-COP* cDNA fragment in the *α1-cop* mutant due to the presence of the T-DNA insertion in the mutant. Similarly, these primers can only amplify the *α1-COP* cDNA fragment and not the *α2-COP* cDNA fragment in the *α2-cop* mutant. As shown in Fig. 3A–C, mRNA levels of *α-COP* were lower in *α2-cop* than in *α1-cop* mutant, which correlates with the growth defects in *α2-cop-3*. On the other hand, the mRNA levels of *α1-COP* and *α2-COP* in *α2-cop-3* and *α1-cop-1-1* mutants, respectively, were similar to those of the wild type (see Supplementary Fig. S3), indicating that there is no expression compensation between the two *α-COP* genes in the mutants. These results therefore suggest that the two α-COP isoforms are differentially expressed and it is the *α2-COP* isoform that contributes most to total *α-COP* levels. The protein levels of α-COP, including both α1 and α2 isoforms, were analyzed by western blot using an antibody against the first 10 amino acids of cow α-COP (Gerich *et al.*, 1995) that has been previously described to recognize Arabidopsis α-COP (Contreras *et al.*, 2004a). Cow α-COP and both Arabidopsis α-COP isoforms share the same first nine aminoacids and so this antibody should recognize both isoforms. As shown in Fig. 3D, the antibody recognized a band of aproximately 130 kDa corresponding to the molecular weight of α-COP. Using this antibody, we found that the *α2-cop-3* mutant also has lower α-COP protein levels than wild type and the *α1-cop* mutant (Fig. 3D). No specific bands from the translation of truncated transcripts were detected in the mutants (Supplemental Fig. S1).

### The loss of function of *α2-COP* affects p24δ5 trafficking and the integrity of the Golgi apparatus

**Fig. 4.**
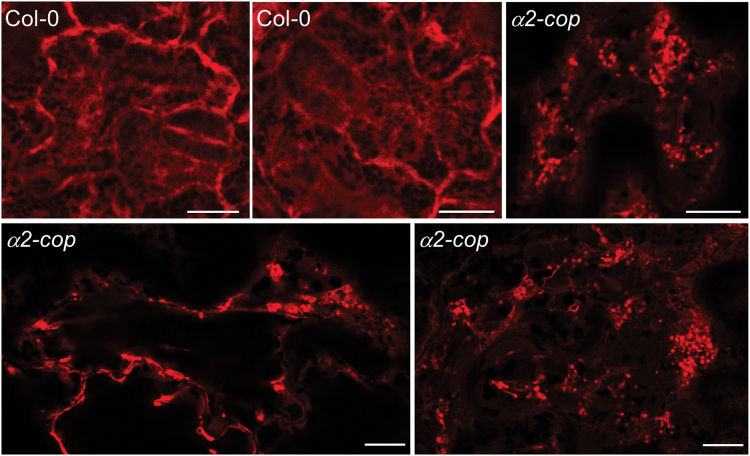
*α2-cop-3* mutant shows abnormal distribution of RFP- p24δ5. Confocal laser scanning microscopy of epidermal cells of 4.5-DAG cotyledons. RFP-p24δ5 mainly localized to the ER network in wild type plants (Col-0). In contrast, it was mostly found in punctate structures, which often appeared in clusters, in the *α2-cop* mutant. Scale bars, 10 µm.

**Fig. 5.**
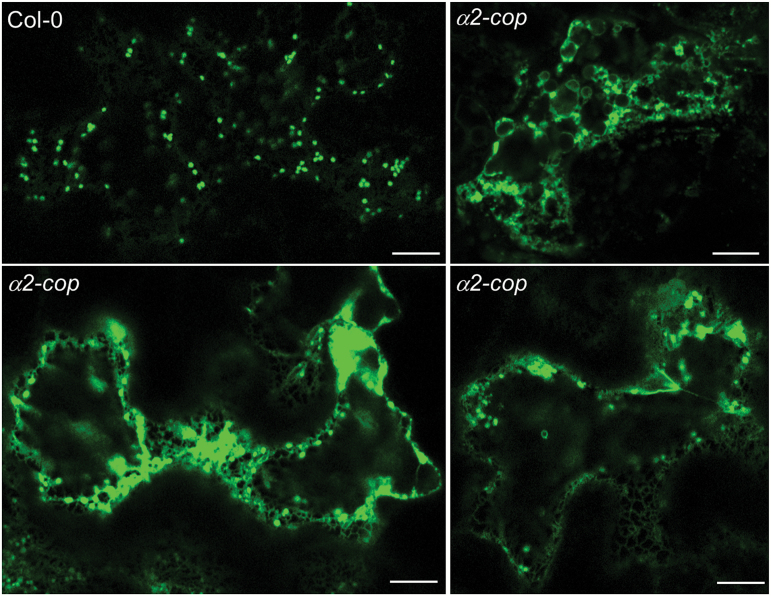
*α2-cop-3* mutant shows abnormal distribution of the Golgi marker ST-YFP. Confocal laser scanning microscopy of epidermal cells of cotyledons 4.5 days after germination. The Golgi marker ST-YFP partially localised to the ER network and to clusters of punctate structures in the *a2-cop-3* mutant. Scale bar, 10 µm.

As the *α2-cop* mutant has defects in growth, we aimed to study if retrograde transport in the early secretory pathway was impaired in this mutant. COPI vesicles are involved in the traffic of some transmembrane proteins that constitutively cycle between the ER and the Golgi using the COPII and COPI systems, such as members of the p24 family. p24 proteins constitute a family of type I transmembrane proteins of approximately 24 kDa present on the membranes of the early secretory pathway (Pastor-Cantizano *et al.*, 2016). We have previously shown that the C-terminal cytosolic tail of the Arabidopsis p24δ subfamily proteins has the ability to interact with ARF1 and coatomer subunits through a dilysine motif and with COPII subunits through a diaromatic motif (Contreras *et al.*, 2004a, 2004b). Using a fluorescence-tagged version of one member of the p24 family, RFP-p24δ5, we have also shown that p24δ5 localizes to the ER at steady state as a consequence of highly efficient COPI-based recycling from the Golgi apparatus and that the dilysine motif is necessary and sufficient for ER localization (Contreras *et al.*, 2004a, 2004b; Langhans *et al.*, 2008; Montesinos *et al.*, 2012). More recently, we have found that p24δ5 interacts with ARF1 and COPI subunits at an acidic pH, consistent with this interaction taking place in the Golgi apparatus (Montesinos *et al.*, 2014). We therefore used p24δ5 as a model protein to study COPI-dependent retrograde Golgi-to-ER trafficking in the *α2-cop* mutant. In wild type plants, RFP-p24δ5 mostly localized to the ER network (Fig. 4), as described previously (Sancho-Andrés *et al*, 2016). In contrast, RFP-p24δ5 localized only partially to the ER and was mostly found in punctate structures, which often appeared in clusters, in the *α2-cop-3* mutant (Fig. 4). Next, we checked the distribution of sialyl transferase-YFP (ST-YFP), a specific membrane marker for plant Golgi (Boevink *et al.*, 1998), in the *α2-cop-3* mutant. As shown in Fig. 5, ST-YFP showed the typical punctate pattern characteristic of normal Golgi stacks in wild type plants. However, in the *α2-cop-3* mutant ST-YFP localized partially to the ER network and to clusters of punctate structures (Fig. 5).

**Fig. 6.**
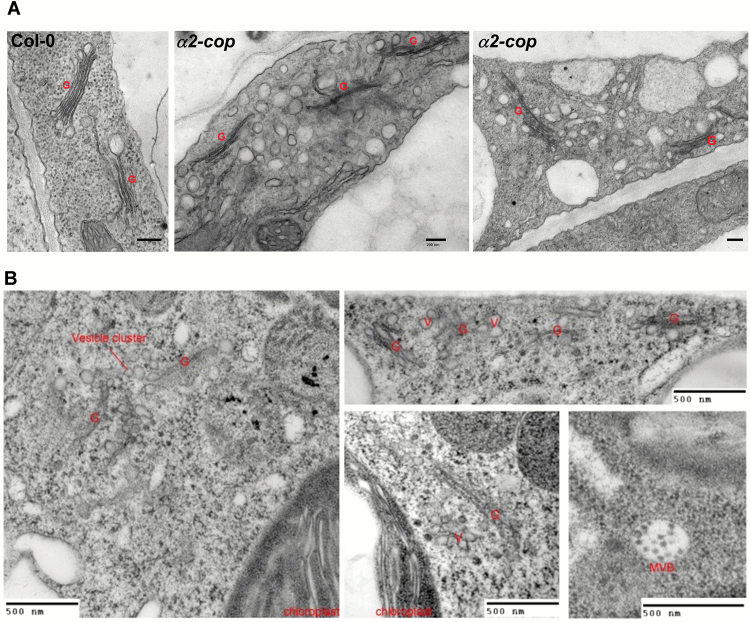
Alteration of Golgi morphology of cotyledon cells in the *α2-cop-3* mutant. A. Chemically fixed cotyledon cells from 4-day-old wild type (Col-0) or *α2-cop* mutant seedlings. Scale bars, 200 nm. B. High-pressure frozen cotyledon cells from from 4-day-old *α2-cop* mutant seedlings. G, Golgi; V, vesicle; MVB, multivesicular body. Scale bars, 500 nm.

To gain insight into the defects observed in the *α2-cop*-3 mutant at the ultrastructural level, we performed transmission electron microscopic (TEM) analysis of ultrathin sections of seedlings processed either by chemical fixation (Fig. 6A) or high pressure freezing/freeze substitution (Fig. 6B). The *α2-cop* mutant showed clear changes in the Golgi apparatus, which in most cases had a reduced number of cisternae per Golgi stack. In addition, the *α2-cop* mutant also contained many abnormal vesicle clusters around the Golgi remnants.

### Transcriptomic analysis of *α2-cop* mutant

**Fig. 7.**
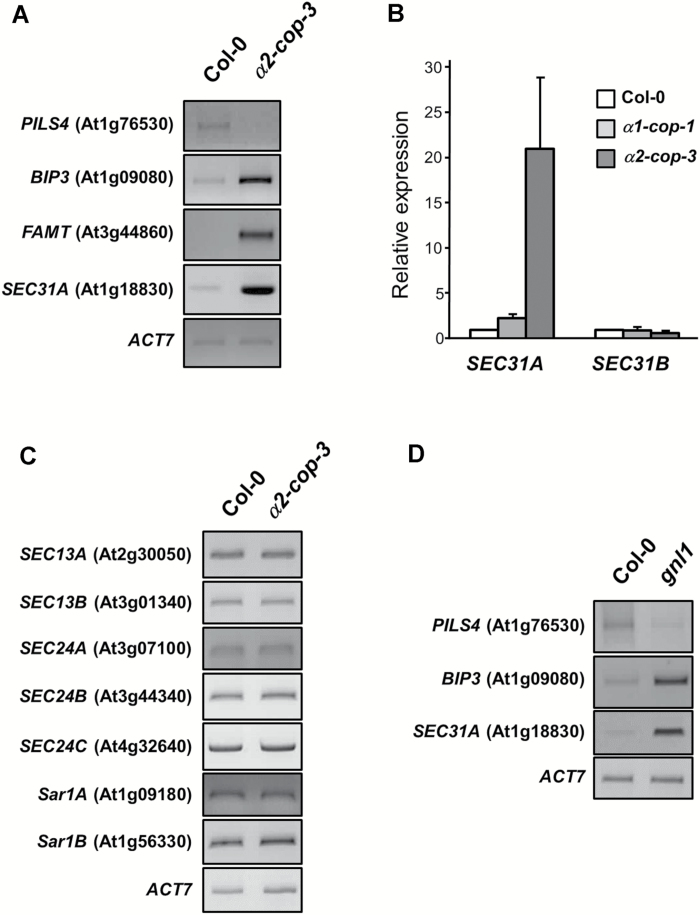
Expression of specific genes in *α1-cop-1*, *α2-cop-3* and *gnl1* mutants. A. sqRT-PCR validation of the microarray data performed for four genes whose expression changed in the *α2-cop-3* mutant. Total RNA was extracted from 4-day-old seedlings. Specific primers were used and *ACT7* was used as a control. B. RT-qPCR analysis of *SEC31A* and *SEC31B* expression in *α1-cop-1* and *α2-cop-3* mutants. Total RNA was extracted from 7-day-old seedlings. The mRNA was analyzed by RT-qPCR with specific primers and normalized to *UBQ10* expression. Results are from two biological samples and three technical replicates. mRNA levels are expressed as relative expression levels and represent fold changes of mutant/wild type. Values represent mean ± SE of the two biological samples. C. sqRT-PCR analysis of COPII subunit genes in the *α2-cop-3* mutant. Total RNA was extracted from 4-day-old seedlings. Specific primers were used and *ACT7* was used as a control. D. sqRT-PCR analysis of *SEC31A*, *BIP3* and *PILS4*, which showed altered expression in *α2-cop-3*, in *gnl1* (SALK_091078C). Total RNA was extracted from 4-day-old seedlings. Specific primers were used and *ACT7* was used as a control. The pattern of expression of all three genes is similar in both *α2-cop-3* and *gnl1*. All specific primers are shown in Supplementary Table S1.

Comparative gene expression analyses were performed to gain insights into the molecular phenotype of the *α2-cop-3* mutant. Global profiling analysis was carried out from 4-day-old seedlings, when the mutant growth phenotype starts to be visible, to detect early changes in expression. A median log_2_ ratio of 1, namely a two-fold difference in expression, of the four biological replicates was used as cut-off criteria to compare the mutant with wild type plants. We identified 534 differentially expressed genes in the *α2-cop-3* mutant; 353 induced (Supplementary Table S2) and 181 repressed (Supplementary Table S3). Confirmation of microarray data was carried out by RT-PCR in the *α2-cop-3* mutant (Fig. 7A) as well as in the *α2-cop-1* and *α2-cop-2* mutants (see Supplementary Fig. S4). GO analysis was performed by agriGO (Du *et al.*, 2010) and GO terms that were overrepresented among upregulated genes (Supplementary Figs. S5-S7, Supplementary Table S4) and downregulated genes (Supplementary Fig. S8, Supplementary Table S5) were selected. Interestingly, the most significantly overrepresented GO cellular component terms among the upregulated genes were ‘plant-type cell wall’ and ‘endomembrane system’ (Supplementary Tables S4, S6). GO terms significantly overrepresented in the biological process category among the upregulated genes were ‘lipid transport’ and ‘cell wall modification’, while one of the terms overrepresented in the molecular function category was ‘oligopeptide transport activity’ (Supplementary Table S4).

Interestingly, one of the genes highly upregulated was *SEC31A*, which encodes one subunit of the COPII coat. The Arabidopsis genome encodes two SEC31 isoforms, SEC31A (At1g18830) and SEC31B (At3g63460). Confirming the microarray data, RT-PCR analysis indicated that the expression of *SEC31A* in the *α2-cop-3* mutant is more than 10 times higher than in wild type (Fig. 7B). *SEC31A* expression was also increased two-fold in the *α1-cop-1* mutant. On the other hand, *SEC31B* expression was not upregulated in either *α2-cop-3* or *α1-cop-1* mutants. This strong upregulation of *SEC31A* in the *α2-cop-3* mutant is consistent with COPII machinery being transcriptionally regulated by the alteration in COPI traffic. However in the *α2-cop-3* mutant this upregulation of *SEC31A* seems to be specific for this particular COPII subunit as the expression other COPII subunit genes did not change (Fig. 7C, Supplementary Tables S2, S3). Finally, we also analyzed the expression of *SEC31A* in *gnl1* (SALK_091078C), a loss-of-function mutant for the ADP-ribosylation factor guanine nucleotide exchange factor (ARF-GEF) GNL1 that regulates COPI formation (Richter *et al.*, 2007; Teh and Moore, 2007; Nakano *et al.*, 2009; Du *et al.*, 2013). Fig. 7D shows that the patterns of activation and repression for some genes that were used to confirm the microarray data of the *α2-cop-3* mutant, were similar in the *gnl1* mutant. This included increased expression of *SEC31A*, suggesting a correlation between the alteration in COPI function and changes in the expression of the COPII subunit *SEC31A*.

## Discussion

In mammals and yeast there is only one isoform of the COPI subunit α-COP. In contrast, two α-COP isoforms, α1- and α2-COP, have been identified in Arabidopsis. Here we have shown that a knockout T-DNA mutant of *α1-COP* resembled wild type plants under standard growth conditions. However, all *α2-COP* T-DNA mutants characterized had defects in root, stem and leaf growth. They were all fertile but short and bushy. The two α-COP isoforms contain at their N-terminal the WD40 domain, which it is required for KKXX-dependent trafficking. It cannot be ruled out that a truncated α-COP protein might be synthesized in the mutants that could account for the difference in the phenotypes between *α1-COP* and *α2-COP* mutants, although no truncated proteins were detected with an N-terminal antibody by western blot. On the other hand, as these isoforms share 93% amino acid sequence identity, the absence of growth defects in the *α1-cop* mutant might be explained by the relative expression levels of both α-COP isoforms. Nevertheless, it cannot be discounted that α2-COP may have specific functions that cannot be performed by α1-COP. Further studies will be required to clarify the differences between the functions of α1-COP and α2-COP. In this study we focused on characterizing the *α2-cop-3* mutant. As the expression of α2-COP-HA in the *α2-cop-3* mutant fully rescued its developmental defects, the results presented here indicate that α2-COP has a role in plant growth.

COPI vesicles are involved in the retrieval of ER-resident proteins from the Golgi apparatus to the ER and also in the traffic of other transmembrane proteins found in the early secretory pathway that are continuously cycling between the ER and Golgi, as is the case for p24 family proteins. Arabidopsis p24δ proteins contain in their cytosolic C-terminus both a dilysine motif in the -3, -4 position, which interacts with COPI via α-COP and β′-COP subunits (Jackson *et al.*, 2012; Eugster, 2004), and a diaromatic motif in the -7, -8 position involved in COPII binding (Contreras *et al.*, 2004b). At steady state, p24δ5 mainly localizes to the ER as a consequence of its highly efficient COPI-based recycling from the Golgi apparatus (Langhans *et al.*, 2008; Montesinos *et al.*, 2012; 2013; 2014; Sancho-Andrés *et al.* 2016). Here, we found that loss of *α2-COP* causes obvious defects in trafficking of RFP-p24δ5, which mostly localized to clusters of punctate structures and was only partially found in the ER network. This probably reflects the inability of p24δ5 to enter standard COPI vesicles for its Golgi to ER retrograde transport. In addition, the localization of the Golgi marker ST-YFP was also altered, which might be the result of fragmentation of the Golgi apparatus, consistent with recent reports showing that silencing of ε-COP and δ-COP in Arabidopsis and β′-COP in *N. benthamiana* results in disruption of the Golgi structure (Ahn *et al.*, 2015; Woo *et al.*, 2015). Indeed, the ultrastructural studies of the *α2-cop-3* mutant revealed severe morphological changes in the Golgi apparatus. These results confirm the role of COPI in mantaining normal cellular function and organelle morphology in the plant early secretory pathway, as previously described in mammals and yeast (Guo *et al.*, 1994; Gaynor and Emr, 1997; Styers *et al.*, 2008).

Results from the microarray revealed upregulation of plant cell wall and endomembrane system genes. As most of these genes encoded proteins that follow or regulate the secretory pathway, this change in gene expression could be a mechanism to compensate for failures in the secretory pathway of the mutant due to the absence of α2-COP. Interestingly, one of the upregulated genes in the *α2-cop-3* mutant was the *SEC31A* gene, which encodes one of the two COPII SEC31 isoforms of Arabidopsis. No changes in COPII subunits other than SEC31A have been detected. SEC31A shows 61% amino acid sequence identity with SEC31B and according to public microarray data (Zimmermann *et al.*, 2004) they are expressed differently in Arabidopsis tissues, with *SEC31B* expression being about 10 times higher than that of *SEC31A*. It has been reported that SEC31B is not able to complement the secretion defect of the *sec31-1* mutant in yeast (De Craene *et al.*, 2014). In that study, SEC31A was not tested and the authors concluded that SEC31A could be the one that complements the secretion defect of the *sec31-1* mutant. In mammals, two SEC31 isoforms have also been identified but their specific roles have not yet been defined. On the other hand, there is evidence that SEC31 interacts directly with SAR1, a small GTPase that controls COPII vesicle biogenesis, to promote SEC23 GTPase Activating Protein activity (Bi *et al.*, 2007). It has been proposed that differences in the affinity for SEC31 between mammalian paralogs of SAR1 together with changes in the stimulated rate of GTP hydrolysis may together cooperate with the intrinsic flexibility of the outer cage in determining COPII vesicle size during assembly and disassembly of the coat on a growing bud (Zanetti *et al.*, 2011). There is increasing evidence that indicates specific expression patterns in COPII subunit isoforms in Arabidopsis may reflect functional diversity (Chung *et al*, 2016). Since the expression of *SEC31A* was highly increased in the *α2-cop* mutant, SEC31A could compete with SEC31B for SAR1 binding resulting in changes in the process of assembly and disassembly of the coat that could adapt ER export machinery under these conditions. The induction of SEC31A might enable efficient packaging of specific cargo proteins into anterograde vesicles or simply increase the overall capacity of anterograde transport to compensate for the effects of the inhibition of retrogade transport in the *α2-cop* mutant. Interestingly, *SEC31A* is also upregulated in the unfolded protein response mediated by the inositol requiring enzyme-1 (IRE1), a response that is known to result in a specific remodeling of the secretory pathway (Nagashima *et al.*, 2011; Song *et al.* 2015). Finally, we also found that *SEC31A* is also strongly upregulated in *gnl1*, a mutant of the ARF-GEF GNL1 involved in COPI assembly. These data suggest that the increase in *SEC31A* expression might be part of a general response to alterations in the secretory pathway.

## Data Deposition

Raw microarray data. Gene Expression Omnibus (GEO). Accession number GSE81049.

https://www.ncbi.nlm.nih.gov/geo/query/acc.cgi?acc=GSE81049

## Supplementary Material

Supplementary Data

## Abbreviations:

ARF1: ADP-ribosylation factor 1
COP: coat protein
ECL: enhanced chemiluminescence
EMPs: endomembrane proteins
GEF: guanine nucleotide exchange factor
GO: gene ontology
HA: human influenza hemagglutinin
MS: Murashige Skoog
RT-PCR: reverse transcription PCR
qPCR: quantitative PCR
ST: sialyl transferase
sqPCRs: semi-quantitative PCRs
TEM: transmission electron microscopic.
