# Supplementary material for: α2-COP is involved in early secretory traffic in Arabidopsis and is required for plant growth
Source: J Exp Bot. 2016 Dec 26;68(3):391–401. doi: 10.1093/jxb/erw446 (PMC5441910; doi:10.1093/jxb/erw446)
Supplement: Supplementary Data [file erw446_Supplementary_Data.zip › Supplementary_Tables_S1_S4_S5.pdf]

α2-COP is involved in early secretory traffic in *Arabidopsis* and is required for plant growth

Fátima Gimeno-Ferrer[1]*, Noelia Pastor-Cantizano[1]*, César Bernat-Silvestre[1]*, Pilar Selvi-Martinez[1], Francisco Vera-Sirera[3], Caiji Gao[2], Miguel Angel Perez-Amador[3], Liwen Jiang[2], Fernando Aniento[1#] and María Jesús Marcote[1#]

*These authors contributed equally to the work

SUPPLEMENTARY TABLES S1, S4 and S5

Supplementary Table S1. Primers used in this study.

Supplementary Table S4. Non-redundant gene ontology (GO) categories significantly overrepresented among up-regulated genes in the *α2-cop-3* mutant.

Supplementary Table S5. Non-redundant gene ontology (GO) biological process categories significantly overrepresented among the down-regulated genes in the *α2-cop-3* mutant.

Supplementary Table S1. Primers used in this study

Name	Purpose	Target	Sequence (5' to 3')
5-alfa1	qPCR	α1-COP At1g62020	GTACACATGAGAACTTTAGCTTCTTGC
3-alfa1	qPCR	α1-COP At1g62020	CAAGTTATCCAGTGCTACTATGCATCA
5-alfa2	qPCR	α2-COP At2g21390	GGCTTGCTATGCTCTGCCTCTCA
3-alfa2	qPCR	α2-COP At2g21390	ACGATTGAAGCACATAATCATTCTGAC
Sec31AIF	qPCR	Sec31A At1g18830	AACGTGATTTTGGTGCAGCGTTA
Sec31AR	qPCR RT-PCR	Sec31A At1g18830	TGGAAGCCAAGAACTGCACTCATC
Sec31BF	qPCR	Sec31B At3g63460	CAGCAGCTGGACCCATAGGATTTAC
Sec31BR	qPCR	Sec31B At3g63460	GCTGTGTTGGAGGACTTGCTGGTTG
UBQ10F	qPCR	UBQ10 At4g05320	GGCCTTGTATAATCCCTGATGAATAAG
UBQ10R	qPCR	UBQ10 At4g05320	AAAGAGATAACAGGAACGGAAACATAGT
Act3	RT-PCR	ACT7 At5g09810	GGAAAACTCACCACCACGAACCAG
Act5	RT-PCR	ACT7 At5g09810	GGATCCAAATGGCCGATGGTGAGG
α125	RT-PCR	α1-COP At1g62020 α2-COP At2g21390	GATTCTGGATTCTTGCAGTTCACC
α123	RT-PCR	α1-COP At1g62020 α2-COP At2g21390	CAGTGAGTGAAGTAAGCAGCGAG
LPα1	Genotyping RT-PCR *α1-COP-1*	α1-COP At1g62020	AGAATTACCTTGGCGAAGAGC
RPα1	Genotyping RT-PCR *α1-COP-1*	α1-COP At1g62020	GGATCCGTGCCATTATCGTTGAGAGATT
RPGα2	Genotyping *α2-COP-3* RT-PCR *α2-COP-2* *α2-COP-3*	α2-COP At2g21390	GCGTACCAGCAGACAAAGAAC

LPGα2	Genotyping<br>*α2-COP-3*<br>RT-PCR<br>*α2-COP-2*<br>*α2-COP-3*	α2-COP<br>At2g21390	GGATCCCATAATCATTCTGACTTGT
NRPα2	Genotyping<br>*α2-COP-1*<br>*α2-COP-2*<br>RT-PCR<br>*α2-COP-1*	α2-COP<br>At2g21390	GGATCCTGTTTCTGTTTCGTAATCTGTG
RPα2-8	Genotyping<br>*α2-COP-1*	α2-COP<br>At2g21390	ATACCATTCATACGCCACAGC
LPα2	Genotyping<br>*α2-COP-2*<br>RT-PCR<br>*α2-COP-1*	α2-COP<br>At2g21390	AGTGCATTGTGAAACTGACCC
BIP35	RT-PCR	BIP3<br>At1g09080	CTGATATCGACGAGATTGTTCTTG
BIP33	RT-PCR	BIP3<br>At1g09080	ATCCTATAGCTAACCGACTC
F5	RT-PCR	FAMT<br>At3g44860	GGATCCATCACAAGACCAGAGTTA
F3	RT-PCR	FAMT<br>At3g44860	GGATCCGACATCACTGAGGCCTCAAG
PILS43	RT-PCR	PILS4<br>At1g76530	GGATCCGTCACAAGCCACATGAAGAATG
PILS45	RT-PCR	PILS4<br>At1g76530	GGATCCTGGAAGTAGAGATGAAGC
SAR1A5	RT-PCR	Sar1A	ATCCGATATCAGGAAGAAGACG
SAR1A3	RT-PCR	Sar1A	GAAGCAAATCACCAATGCTGT
SAR1B5	RT-PCR	Sar1B	GAAGAAGACGAAATCACGAAACC
SAR1B3	RT-PCR	Sar1B	GGTGGTCTAACTTGGGAATAATG
SEC13A5	RT-PCR	Sec13A	GGACAATCATTTCGGAAACATGCCA
SEC13A3	RT-PCR	Sec13A	GGATCCCAGTGTGCTTTTGAAGAG
SEC13B51	RT-PCR	Sec13B	TGAAAGCATTCAGGAAACAT
SEC13B32	RT-PCR	Sec13B	ACAGGTCCCTAGAAATTGTATACAGAA
SEC24A5	RT-PCR	Sec24A	GTAAGTGATAAGTTGTGATCAATAATG
SEC24A3	RT-PCR	Sec24A	ATGGCGGTGGAGGTCTACT
NRPSEC24B	RT-PCR	Sec24B	GATAGAAGATCAACAGTGCATGGGA
NLPSEC24B	RT-PCR	Sec24B	GTTCTCCTCCACCTATGGGA
RPSEC24C	RT-PCR	Sec24C	TTGCAAGCAGCGGCAGTAGCAC

LPSEC24C	RT-PCR	Sec24C	AGGATTTGATCTCCATCTCGTA
Sec31A5	RT-PCR	Sec31A<br>At1g18830	CTCCTCCAGTCCGACCTATGACTC
LBb1	Genotyping<br>Salk lines	T-DNA	GGATCCGCGTGGACCGCTTGCTGCAACT
O-8409	Genotyping<br>Gabi-kat lines	T-DNA	ATATTGACCATCATACTCATTGC

Supplementary Table S4. Non-redundant gene ontology (GO) categories significantly overrepresented among up-regulated genes in the *α2-cop-3* mutant.

Biological Process

Description	GO Term	Number in input list (343 genes)*	Percentage input list	Number in Reference (37767 genes)	Percentage Reference	p-value	FDR
response to hormone stimulus	GO:0009725	23	6,7	982	2,6	4,9E-05	3,5E-03
response to external stimulus	GO:0009605	13	3,8	429	1,1	2,1E-04	1,1E-02
protein amino acid phosphorylation	GO:0006468	21	6,1	946	2,5	2,1E-04	1,1E-02
defense response	GO:0006952	18	5,2	766	2,0	3,1E-04	1,4E-02
response to oxidative stress	GO:0006979	11	3,2	332	0,9	3,1E-04	1,4E-02
oligopeptide transport	GO:0006857	5	1,5	68	0,2	5,2E-04	2,1E-02
response to bacterium	GO:0009617	9	2,6	247	0,7	5,7E-04	2,1E-02
response to chitin	GO:0010200	7	2,0	151	0,4	6,0E-04	2,2E-02
lipid transport	GO:0006869	7	2,0	163	0,4	9,3E-04	3,2E-02
cell wall modification	GO:0042545	6	1,7	123	0,3	1,1E-03	3,6E-02

Cellular component

Description	GO Term	Number in input list (343 genes)	Percentage input list	Number in Reference (37767 genes)	Percentage Reference	p-value	FDR
plant-type cell wall	GO:0009505	13	3,8	254	0,7	1,10E-06	1,30E-04
endomembrane system	GO:0012505	53	15,5	3416	9,0	1,00E-04	3,00E-03

Molecular Function

Description	GO Term	Number in input list (343 genes)*	Percentage input list	Number in Reference (37767 genes)	Percentage Reference	p-value	FDR
oligopeptide transporter activity	GO:0015198	5	1,5	21	0,1	3,2E-06	5,6E-04
protein kinase activity	GO:0004672	21	6,1	953	2,5	2,3E-04	1,6E-02
enzyme inhibitor activity	GO:0004857	7	2,0	173	0,5	1,3E-03	3,8E-02
sugar:hydrogen symporter activity	GO:0005351	6	1,7	126	0,3	1,3E-03	3,8E-02
peroxidase activity	GO:0004601	6	1,7	127	0,3	1,3E-03	3,8E-02
electron carrier activity	GO:0009055	9	2,6	294	0,8	1,8E-03	4,8E-02

*** 343 genes with valid annotation**

Supplementary Table S5. Non-redundant gene ontology (GO) biological process categories significantly overrepresented among the down-regulated genes in the *α2-cop-3* mutant.

Biological Process

Description	GO Term	Number in input list (178 genes)*	Percentage input list	Number in Reference (37767 genes)	Percentage Reference	p-value	FDR
response to heat	GO:0009408	10	5,5	161	0,4	9,4E-09	2,3E-06
response to UV-B	GO:0010224	5	2,8	48	0,1	5,1E-06	3,1E-04
response to hydrogen peroxide	GO:0042542	5	2,8	53	0,1	8,0E-06	4,3E-04
response to high light intensity	GO:0009644	5	2,8	57	0,2	1,1E-05	4,9E-04
phenylpropanoid biosynthetic process	GO:0009699	6	3,3	141	0,4	7,2E-05	2,5E-03
response to jasmonic acid stimulus	GO:0009753	7	3,9	215	0,6	9,1E-05	2,7E-03
response to salt stress	GO:0009651	8	4,4	366	1,0	4,1E-04	1,0E-02
response to gibberellin stimulus	GO:0009739	5	2,8	159	0,4	1,1E-03	2,3E-02
regulation of cellular biosynthetic process	GO:0031326	19	10,5	1881	5,0	1,6E-03	3,1E-02
lipid metabolic process	GO:0006629	11	6,1	841	2,2	2,4E-03	4,0E-02
response to abscisic acid stimulus	GO:0009737	7	3,9	378	1,0	2,4E-03	4,0E-02
response to salicylic acid stimulus	GO:0009751	5	2,8	200	0,5	2,9E-03	4,4E-02
response to ethylene stimulus	GO:0009723	5	2,8	199	0,5	2,8E-03	4,4E-02
regulation of gene expression	GO:0010468	19	10,5	2001	5,3	3,1E-03	4,5E-02

Cellular Component

No significant GO categories found

Molecular Function

No significant GO categories found

*** 178 genes with valid annotation**
