# Supplementary material for: α2-COP is involved in early secretory traffic in Arabidopsis and is required for plant growth
Source: J Exp Bot. 2016 Dec 26;68(3):391–401. doi: 10.1093/jxb/erw446 (PMC5441910; doi:10.1093/jxb/erw446)
Supplement: Supplementary Data [file erw446_Supplementary_Data.zip › Supplementary_Figures_S1_S8.pdf]

α2-COP is involved in early secretory traffic in *Arabidopsis* and is required for plant growth

Fátima Gimeno-Ferrer[1]*, Noelia Pastor-Cantizano[1]*, César Bernat-Silvestre[1]*, Pilar Selvi-Martinez[1], Francisco Vera-Sirera[3], Caiji Gao[2], Miguel Angel Perez-Amador[3], Liwen Jiang[2], Fernando Aniento[1#] and María Jesús Marcote[1#]

*****These authors contributed equally to the work

SUPPLEMENTARY FIGURES S1-S8

Supplementary Figure S1

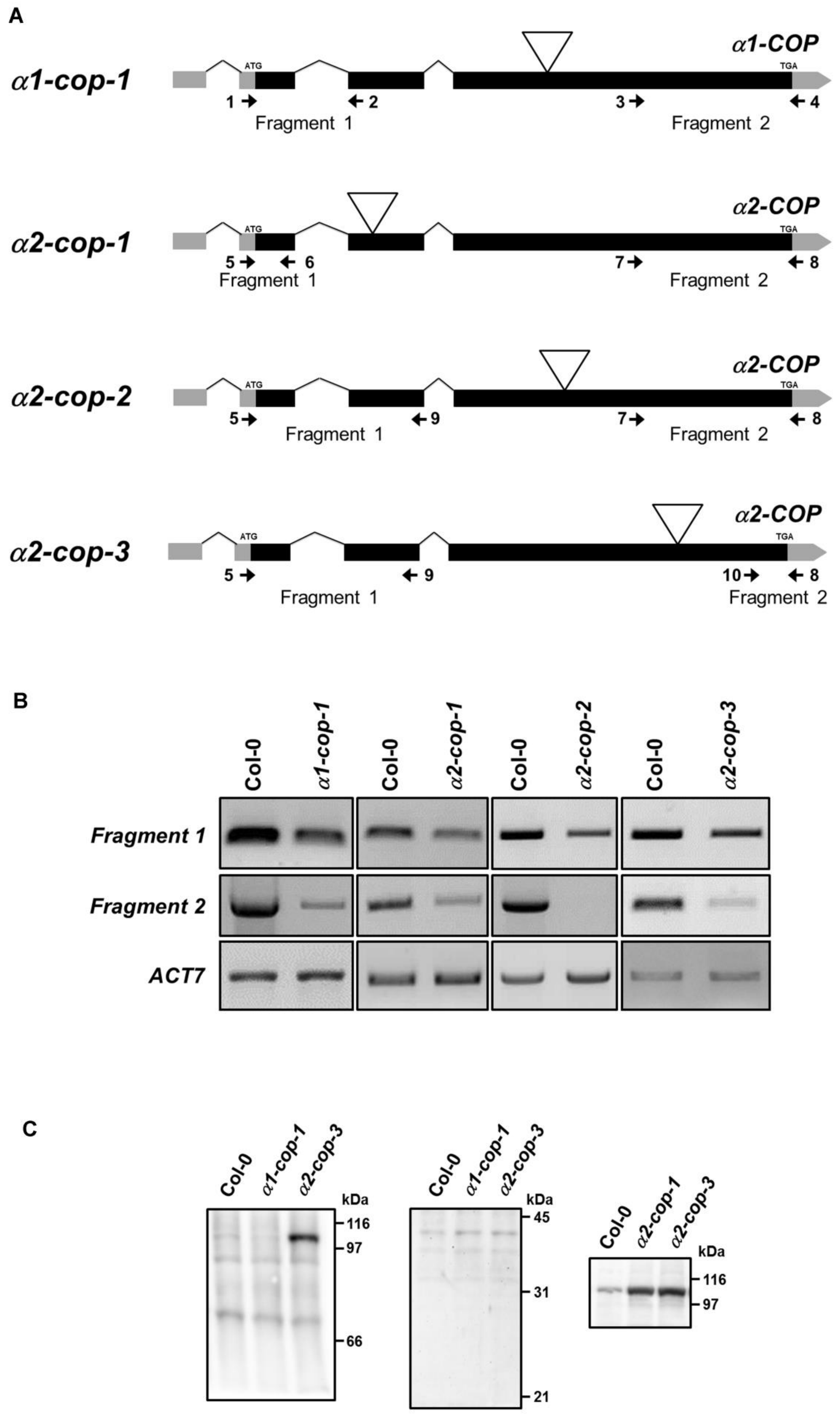

Supplementary Fig. S1. sqRT-PCR and western blot analysis of *α1-cop* and *α2-cop* mutants to detect truncated transcripts and proteins, respectively.

A. Diagram of *α1-COP* and *α2-COP* genes and localization of the T-DNA insertion in the mutants. The primers used in the RT-PCR to detect truncated transcripts, up- and downstream of the T-DNA insertion, are pointed out by arrows. Sequences of the primers 1 (RPα1), 4 (3-alfa1), 5 (NRPα2), 7 (RPGα2) and 8 (LPGα2) are in Supplementary Table S1. The other primer sequences are as follows:
2, 5 prime-GTACTCATGATGGAACTGAACCG-3 prime;
3, 5 prime-GCTCTTCGCCAAGGTAATTCT-3 prime;
6, 5 prime-TCGATCAAAGTACCCATACGAT-3 prime;
9, 5 prime-ACAGCTTTACTTGG CGGTCATCG-3 prime;
10, 5 prime-GTGATATGCGGGTCCACTTA-3 prime.

B. Total RNA from 7-day-old seedlings of the mutants and wild type (Col-0) were used for the RT-PCR. For PCRs, gene specific primers (see A) were used. *ACT7* was used as a control.

C. Western blot analysis of total protein extracts from 7-day-old seedlings of wild type, *α1-cop-1*, *α2-cop-1* and *α2-cop-3* mutants using an N-terminal α-COP peptide antibody to detect any N-terminal truncated proteins that may arise from translation of the truncated transcripts detected in B. 10 μg of total protein was loaded in each lane. The expected molecular weight of the truncated proteins if translation occurs up to the T-DNA insertion are approximately 40 kDa for *αl-cop-1*, 10 kDa for *α2-cop-1* and 100 kDa for *α2-cop-3*. The 100 kDa band detected in the *α2-cop-3* mutant cannot be a truncated α2-COP protein as it is also detected in the *α2-cop-1* mutant. This band might correspond to a protein encoded by one of the genes induced in the *α2-cop* mutants that is recognized by the antibody.

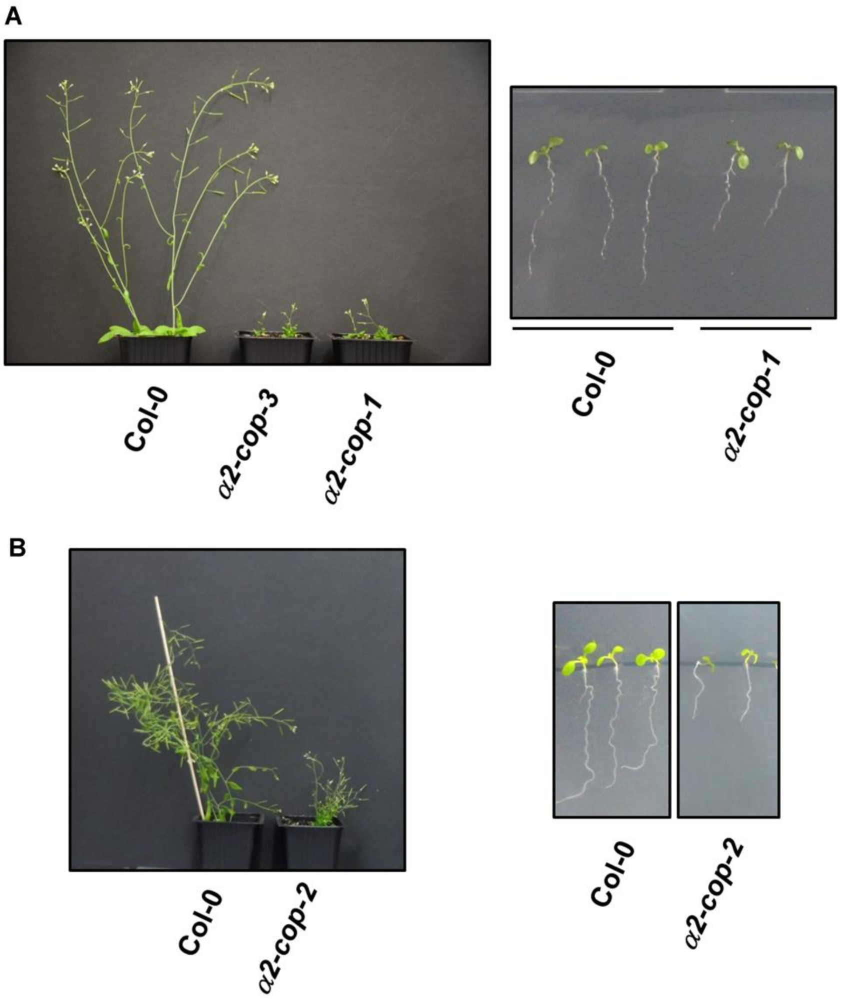

Supplementary Fig. S2. *α2-cop* mutants show the same growth phenotype. A. 40-day-old plants and 7-day-old seedlings of *α2-cop-1*, *α2-cop-3* and wild type (Col-0). B. 50-day-old plants and 7-day-old seedlings of *α2-cop-2* and wild type (Col-0).

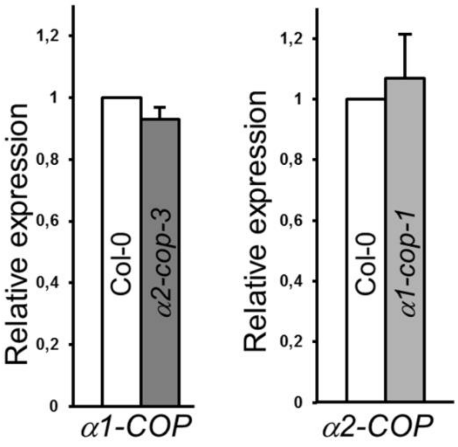

Supplementary Fig. S3. RT-qPCR analysis of *α1-COP* and *α2-COP* expression in *α2-cop-3* and *α1-cop-1* mutants, respectively. Total RNAs were extracted from 7-day-old seedlings. The mRNA was analyzed by RT-qPCR with the *α1-COP* specific primers, 5-alfa1 and 3-alfa1 and the *α2-COP* specific primers, 5-alfa2 and 3-alfa2, and normalized to *UBQ10* expression. Results are from two biological samples and three technical replicates. mRNA levels are expressed as relative expression levels and represent fold changes of mutant/wild type. Values represent mean ± SE of the two biological samples.

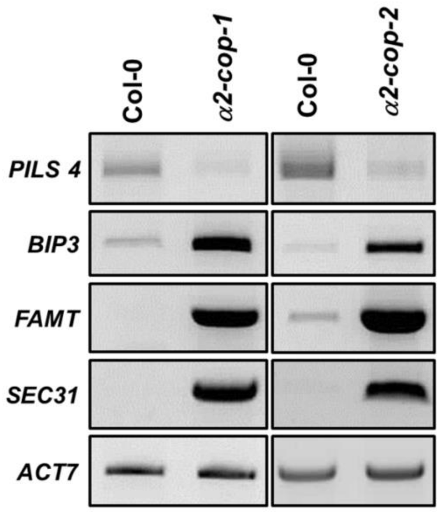

Supplementary Fig. S4. Confirmation of the *α2-cop-3* microarray data in *α2-cop-1* and *α2-cop-2*. sqRT-PCR in *α2-cop-1* and *α2-cop-2* mutants of four genes whose expression changed in the *α2-cop-3* mutant. Total RNAs were extracted from 4-day-old seedlings. Specific primers were used and *ACT7* was used as a control.

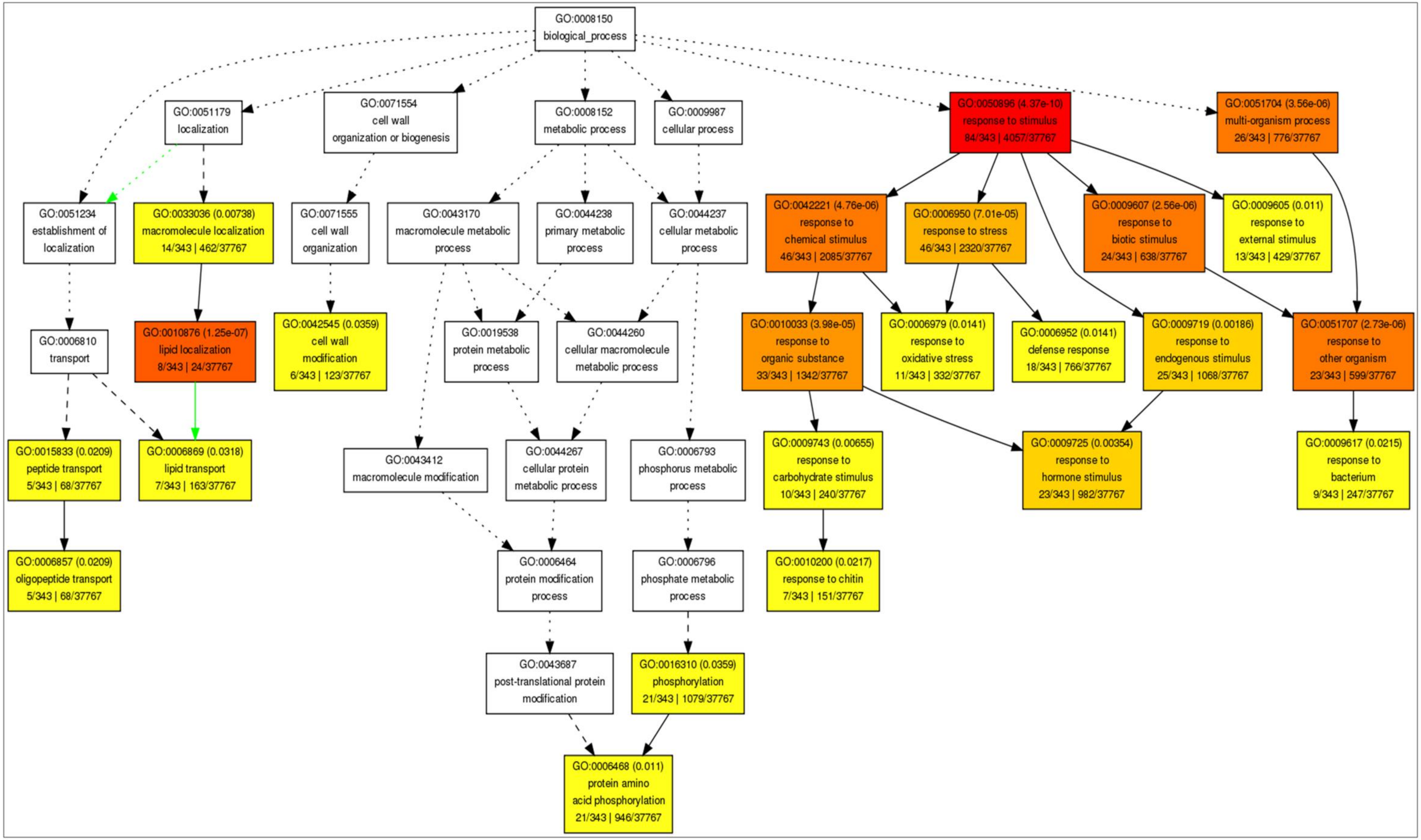

GO:0008150
biological_process
GO:0051179
localization
GO:0071554
cell wall
organization or biogenesis
GO:0008152
metabolic process
GO:0009987
cellular process
GO:0050896 (4.37e-10)
response to stimulus
84/343 | 4057/37767
GO:0051704 (3.56e-06)
multi-organism process
26/343 | 776/37767
GO:0051234
establishment of
localization
GO:0033036 (0.00738)
macromolecule localization
14/343 | 462/37767
GO:0071555
cell wall
organization
GO:0043170
macromolecule metabolic
process
GO:0044238
primary metabolic
process
GO:0044237
cellular metabolic
process
GO:0042221 (4.76e-06)
response to
chemical stimulus
46/343 | 2085/37767
GO:0006950 (7.01e-05)
response to stress
46/343 | 2320/37767
GO:0009607 (2.56e-06)
response to
biotic stimulus
24/343 | 638/37767
GO:0009605 (0.011)
response to
external stimulus
13/343 | 429/37767
GO:0006810
transport
GO:0010876 (1.25e-07)
lipid localization
8/343 | 24/37767
GO:0042545 (0.0359)
cell wall
modification
6/343 | 123/37767
GO:0019538
protein metabolic
process
GO:0044260
cellular macromolecule
metabolic process
GO:0010033 (3.98e-05)
response to
organic substance
33/343 | 1342/37767
GO:0006979 (0.0141)
response to
oxidative stress
11/343 | 332/37767
GO:0006952 (0.0141)
defense response
18/343 | 766/37767
GO:0009719 (0.00186)
response to
endogenous stimulus
25/343 | 1068/37767
GO:0051707 (2.73e-06)
response to
other organism
23/343 | 599/37767
GO:0015833 (0.0209)
peptide transport
5/343 | 68/37767
GO:0006869 (0.0318)
lipid transport
7/343 | 163/37767
GO:0043412
macromolecule modification
GO:0044267
cellular protein
metabolic process
GO:0006793
phosphorus metabolic
process
GO:0009743 (0.00655)
response to
carbohydrate stimulus
10/343 | 240/37767
GO:0009725 (0.00354)
response to
hormone stimulus
23/343 | 982/37767
GO:0009617 (0.0215)
response to
bacterium
9/343 | 247/37767
GO:0006857 (0.0209)
oligopeptide transport
5/343 | 68/37767
GO:0006464
protein modification
process
GO:0006796
phosphate metabolic
process
GO:0010200 (0.0217)
response to chitin
7/343 | 151/37767
GO:0043687
post-translational protein
modification
GO:0016310 (0.0359)
phosphorylation
21/343 | 1079/37767
GO:0006468 (0.011)
protein amino
acid phosphorylation
21/343 | 946/37767

Supplementary Fig. S5. Hierarchical view of gene ontology (GO) categories significantly overrepresented among the upregulated genes in the *α2-cop-3* mutant: Biological process terms. Analysis was carried out using the AgriGO tool (Du *et al.*, 2010). Significant categories were selected with the p-value from Fisher's exact test corrected for multiple hypothesis testing, $P<0.05$. A color scale was used according to the significance level of each GO term.

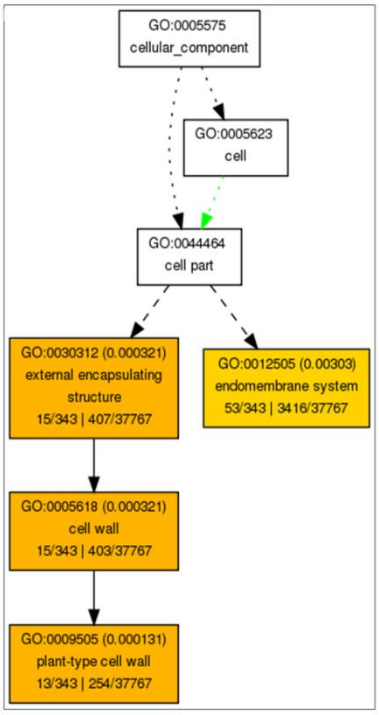

Supplementary Fig. S6. Hierarchical view of gene ontology (GO) categories significantly overrepresented among the upregulated genes in the *α2-cop-3* mutant: Cellular component terms. Analysis was carried out using the AgriGO tool (Du *et al.*, 2010). Significant categories were selected with the p-value from Fisher's exact test corrected for multiple hypothesis testing, *P*<0.05. A color scale was used according to the significance level of each GO term.

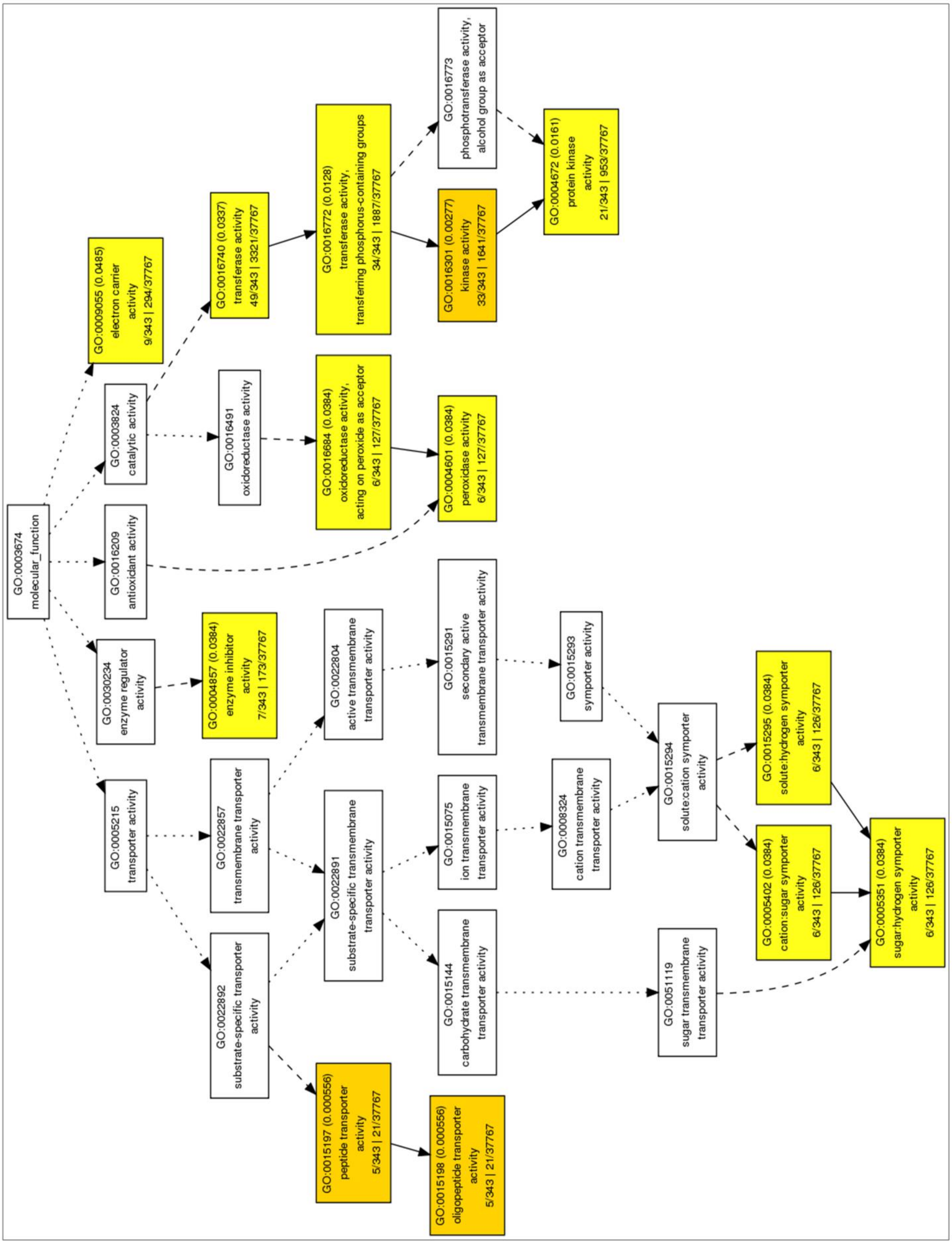

Supplementary Fig. S7. Hierarchical view of gene ontology (GO) categories significantly overrepresented among the upregulated genes in the *α2-cop-3* mutant: Molecular function terms. Analysis was carried out using the AgriGO tool (Du *et al.*, 2010). Significant categories were selected with the p-value from Fisher's exact test corrected for multiple hypothesis testing, $P<0.05$. A color scale was used according to the significance level of each GO term.

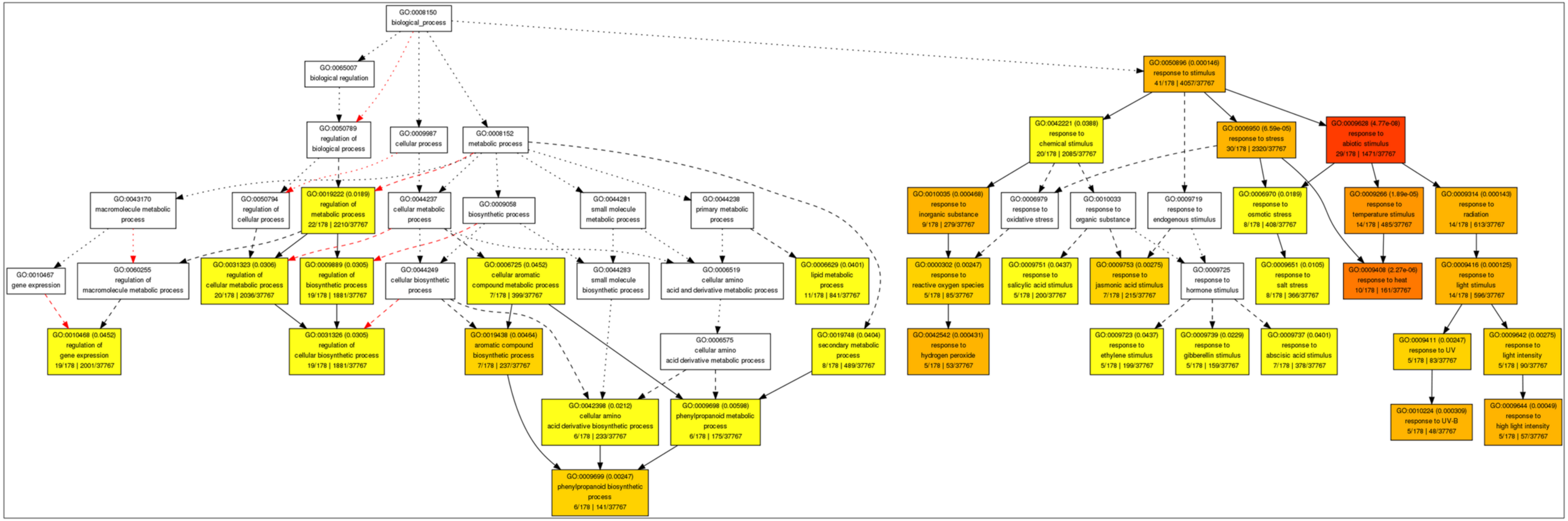
GO:0008150 biological_process
GO:0065007 biological regulation
GO:0050789 regulation of biological process
GO:0009987 cellular process
GO:0008152 metabolic process
GO:0043170 macromolecule metabolic process
GO:0050794 regulation of cellular process
GO:0019222 (0.0189) regulation of metabolic process 22/178 | 2210/37767
GO:0044237 cellular metabolic process
GO:0009058 biosynthetic process
GO:0044281 small molecule metabolic process
GO:0044238 primary metabolic process
GO:0010467 gene expression
GO:0060255 regulation of macromolecule metabolic process
GO:0031323 (0.0306) regulation of cellular metabolic process 20/178 | 2036/37767
GO:0009889 (0.0305) regulation of biosynthetic process 19/178 | 1881/37767
GO:0044249 cellular biosynthetic process
GO:0006725 (0.0452) cellular aromatic compound metabolic process 7/178 | 399/37767
GO:0044283 small molecule biosynthetic process
GO:0006519 cellular amino acid and derivative metabolic process
GO:0006629 (0.0401) lipid metabolic process 11/178 | 841/37767
GO:0010468 (0.0452) regulation of gene expression 19/178 | 2001/37767
GO:0031326 (0.0305) regulation of cellular biosynthetic process 19/178 | 1881/37767
GO:0019438 (0.00464) aromatic compound biosynthetic process 7/178 | 237/37767
GO:0006575 cellular amino acid derivative metabolic process
GO:0019748 (0.0404) secondary metabolic process 8/178 | 489/37767
GO:0042398 (0.0212) cellular amino acid derivative biosynthetic process 6/178 | 233/37767
GO:0009698 (0.00598) phenylpropanoid metabolic process 6/178 | 175/37767
GO:0009699 (0.00247) phenylpropanoid biosynthetic process 6/178 | 141/37767
GO:0050896 (0.000146) response to stimulus 41/178 | 4057/37767
GO:0042221 (0.0388) response to chemical stimulus 20/178 | 2085/37767
GO:0006950 (6.59e-05) response to stress 30/178 | 2320/37767
GO:0009628 (4.77e-08) response to abiotic stimulus 29/178 | 1471/37767
GO:0010035 (0.000468) response to inorganic substance 9/178 | 279/37767
GO:0006979 response to oxidative stress
GO:0010033 response to organic substance
GO:0009719 response to endogenous stimulus
GO:0006970 (0.0189) response to osmotic stress 8/178 | 408/37767
GO:0009266 (1.89e-05) response to temperature stimulus 14/178 | 485/37767
GO:0009314 (0.000143) response to radiation 14/178 | 613/37767
GO:0000302 (0.00247) response to reactive oxygen species 5/178 | 85/37767
GO:0009751 (0.0437) response to salicylic acid stimulus 5/178 | 200/37767
GO:0009753 (0.00275) response to jasmonic acid stimulus 7/178 | 215/37767
GO:0009725 response to hormone stimulus
GO:0009651 (0.0105) response to salt stress 8/178 | 366/37767
GO:0009408 (2.27e-06) response to heat 10/178 | 161/37767
GO:0009416 (0.000125) response to light stimulus 14/178 | 596/37767
GO:0042542 (0.000431) response to hydrogen peroxide 5/178 | 53/37767
GO:0009723 (0.0437) response to ethylene stimulus 5/178 | 199/37767
GO:0009739 (0.0229) response to gibberellin stimulus 5/178 | 159/37767
GO:0009737 (0.0401) response to abscisic acid stimulus 7/178 | 378/37767
GO:0009411 (0.00247) response to UV 5/178 | 83/37767
GO:0009642 (0.00275) response to light intensity 5/178 | 90/37767
GO:0010224 (0.000309) response to UV-B 5/178 | 48/37767
GO:0009644 (0.00049) response to high light intensity 5/178 | 57/37767

Supplementary Fig. S8. Hierarchical view of gene ontology (GO) categories significantly overrepresented among the downregulated genes in the *α2-cop-3* mutant. Analysis was carried out using the AgriGO tool (Du *et al.*, 2010). Significant categories were selected with the p-value from Fisher's exact test corrected for multiple hypothesis testing, $P<0.05$. A color scale was used according to the significance level of each GO term. Only biological process terms were overrepresented.
